# Low-Grade Fibromyxoid Sarcoma of the Lateral Skull Base: Presentation of Two Cases

**DOI:** 10.1155/2019/7917040

**Published:** 2019-07-08

**Authors:** Evgenia Chetverikova, Priit Kasenõmm

**Affiliations:** ^1^Department of Otorhinolaryngology, Tartu University Hospital, J. Kuperjanovi 1, 51003 Tartu, Estonia; ^2^Institute of Clinical Medicine, University of Tartu, L. Puusepa 8, 50406 Tartu, Estonia

## Abstract

Low-grade fibromyxoid sarcoma (LGFMS) is a rare slow-growing malignant tumour with a deceptively benign histologic appearance. Herein, we report two cases of LGFMS with variable clinical presentations. The first case was a 17-year-old female who referred to our department due to deaf ear on the right together with ipsilateral gag reflex impairment and globus sensation in the pharynx. The second case was a 35-year-old female with recurrent LGFMS, suffering from headaches, vertigo, and episodes of loss of consciousness. LGFMS of the temporal bone is a rare pathology, and to the best of our knowledge, no such cases have been reported before.

## 1. Introduction

Head and neck sarcomas account for only 1% of all head and neck malignancies and 5% of all sarcomas [1, 2]. Among them, low-grade fibromyxoid sarcoma (LGFMS) is a rare, slow-growing, malignant soft tissue tumour with a deceptively benign histological appearance [3]. LGFMS occurs most commonly in the deep soft tissues of the proximal extremities and trunk [3], with only isolated head and neck cases reported [4–8]. The tumour typically affects young adults, although children and older adults can also be affected [3–5]. The diagnosis is made based on histopathological examination and supported by immunohistochemical analysis. Herein, we report two cases of lateral skull base LGFMSs with different clinical presentations. To the best of our knowledge, no such cases have been reported previously.

## 2. Case 1

**Figure 1:**
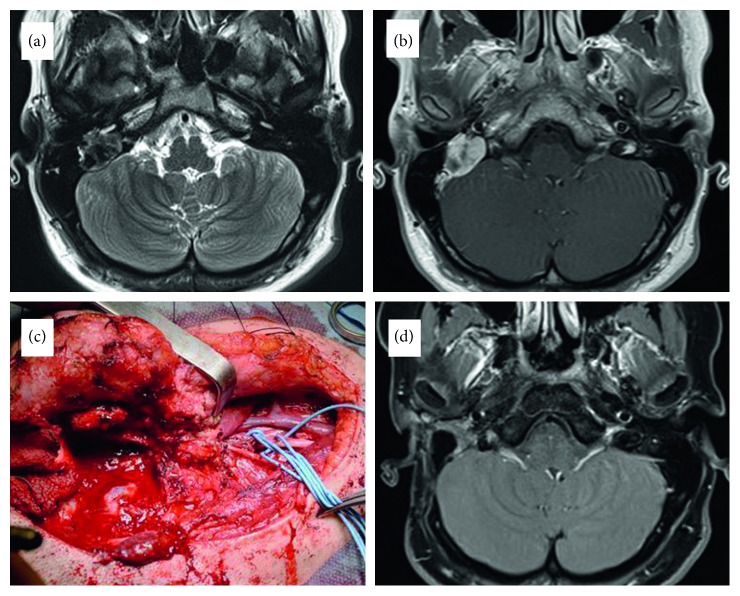
Pre- and postoperative MRI scans of Case 1. (a) Preoperative MRI T2 sequence showing the tumour measuring 25 × 20 mm at the jugular foramen with infralabyrinthine extension causing erosion of the posterior semicircular canal. Note clear distinction between the tumour and cerebellum posteriorly. (b) Preoperative MRI T1 with gadolinium enhancement. (c) Intraoperative picture after extended mastoidectomy and neck dissection. The next step was anterior rerouting of the facial nerve, which gives required access to the jugular foramen. (d) Postoperative MRI T1 with gadolinium enhancement after 3 years of follow-up. Total tumour clearance and no evidences of recurrence could be confirmed.

**Figure 2:**
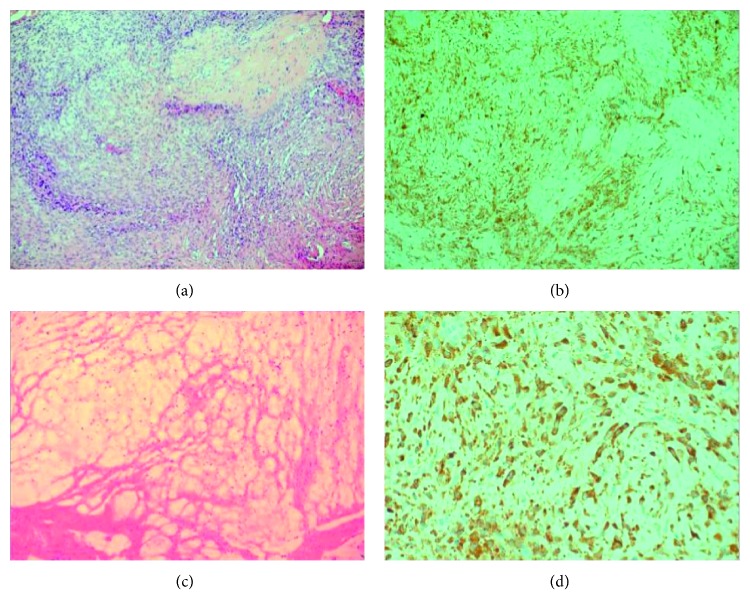
Histopathological features of LGFMS. (a) Fibrous stroma containing myxoid parts and whorling spindle cells (H&E × 100) (b) Tumour cells show diffuse reactivity for MUC4. (c) LGFMS with alternating fibrous and myxoid areas (H&E × 100). (d) Tumour cells show strong cytoplasmic staining for MUC4.

A 17-year-old girl presented with globus sensation in the pharynx and deaf ear on the right side. Clinical examination revealed gag reflex impairment on the right, indicating paralysis of the glossopharyngeal nerve. Otoscopy showed an expansive process of the right middle ear. The function of the facial nerve and other cranial nerves was intact. Otorhinolaryngological findings were otherwise normal. An MRI scan revealed a tumour measuring 25 × 20 mm at the right jugular foramen with extension into middle ear and mastoid, causing involvement of the mastoid segment of the facial nerve and erosion of the posterior semicircular canal (Figure 1). The initial diagnosis included tympanojugular paraganglioma class C2 or endolymphatic sac tumour based on the tumour location and appearance on CT and MRI scans. The surgical excision of the tumour was performed using infratemporal fossa approach type A (ITFA-A) with preoperative embolization. The operation was undertaken in a manner that has been described previously [9], consisting of anterior rerouting of the facial nerve and selective neck dissection levels IIA, IIB, and III. Wide exposure of the tumour locating at the level of the jugular foramen, inferior portion of the labyrinth, infralabyrinthine air cells, and hypotympanum was achieved followed by its piecemeal resection. Histopathology and immunohistochemistry of the tumour tissue revealed LGFMS (Figures 2(a) and 2(b)). Due to malignant nature of this tumour and contaminated (R1) margins, the course of postoperative radiotherapy was undertaken (54 Gy). The postoperative period was otherwise unremarkable, and no recurrence of the disease has been found after 3 years of follow-up using annual MRI scans. The patient has normal facial nerve (House–Brackmann grade 1) and X, XI, and XII cranial nerve functions. Preoperative glossopharyngeal nerve palsy remained unchanged.

## 3. Case 2

**Figure 3:**
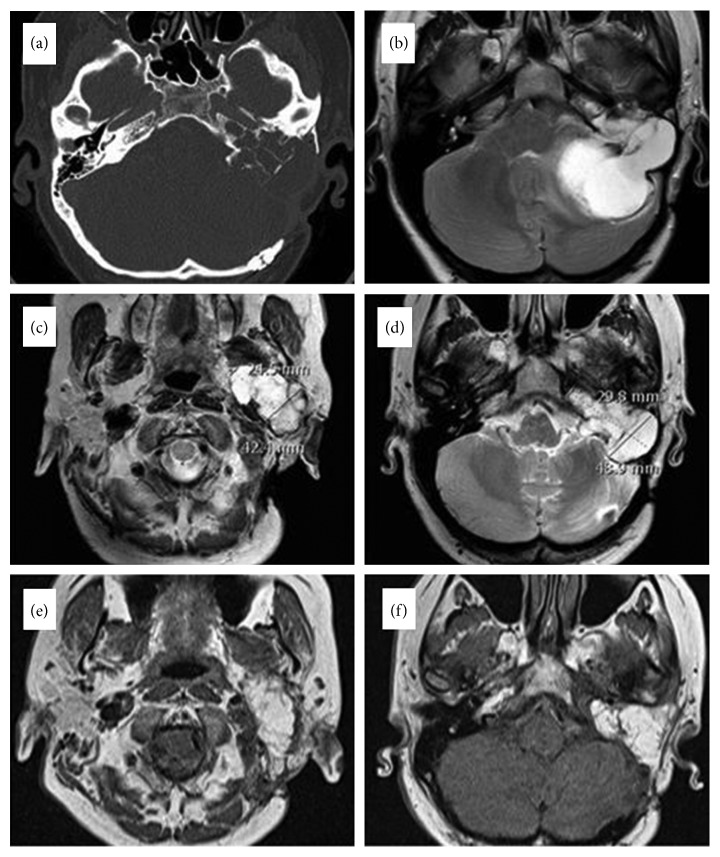
Pre- and postoperative CT and MRI scans of Case 2. (a) Preoperative CT scan showing calcifications within the tumour. (b) Preoperative MRI showing a large arachnoid cyst posteriorly to the tumour at the right cerebellopontine angle. Note remarkable compression of the cerebellum and brainstem caused by the cyst. (c, d) Preoperative MRI scans showing tumour masses in the right parapharyngeal space and at the projection of the temporal bone, respectively. (e, f) Postoperative MRI scans showing fat tissue at the right parapharyngeal space and at the projection of the temporal bone, respectively.

A 33-year-old woman was referred to our department due to recurrent LGFMS of the lateral skull base. She was first diagnosed with LGFMS in 2001 when the patient underwent partial tumour resection using the transtemporal approach with blind-sac closure of the external auditory canal without rerouting of the facial nerve in another hospital. The definite diagnosis of LGFMS was established based on postoperative histopathology and immunohistochemistry (Figures 2(c) and 2(d)). For the next 15 years, the patient was lost to follow-up. In 2016, she was referred to our department suffering from unsteadiness, headaches, episodes of loss of consciousness, and left-sided hearing loss. Her physical examination revealed a slight unsteadiness, a left facial nerve paralysis (House–Brackmann grade 3), and numbness in the ipsilateral side of the face. MRI showed a large arachnoid cyst at the left cerebellopontine angle markedly displacing the brainstem (Figure 3(b)). The MRI scan also revealed two expansive lesions, one at the projection of the left temporal bone measuring 4.9 × 3.0 × 4.4 cm and another in the left parapharyngeal space measuring 4.2 × 2.5 × 3.7 cm (Figures 3(c) and 3(d)). The decision was made to stage the surgery. At first, the patient underwent suboccipital craniotomy for arachnoid cyst resection in order to achieve brainstem decompression. During surgery, biopsy was also taken from the tumour mass, which confirmed the recurrence of LGFMS. The next step was surgical removal of the tumour using the transcochlear approach with selective neck dissection levels IIA, IIB, and III. At surgery, complete resection of the parapharyngeal tumour was performed, followed by extradural subtotal resection of the tumour at the level of the temporal bone. Small part of the tumour infiltrating medial wall of the jugular foramen was preserved in order to keep function of the lower cranial nerves. The entire intratemporal course of the facial nerve was sacrificed and resected together with the tumour. Reconstruction of the facial nerve was performed using cable grafting with the great auricular nerve. The nerve graft was used to achieve the anastomosis between the intraparotid segment and the internal auditory canal segment of the facial nerve. Postoperatively, the patient developed complete left-sided facial nerve paralysis (House–Brackmann grade 6). Histological examination confirmed the diagnosis of LGFMS. The patient was discharged from the hospital and received radiotherapy (48 Gy for the tumour resection line and 68 Gy for residual tissue). Postoperative MRI and CT scans showed almost total tumour clearance except the small residual tumour at the level of the left jugular foramen (Figures 3(e) and 3(f)).

## 4. Discussion

LGFMS most commonly presents as an intramuscular soft tissue mass of the proximal extremities or the trunk [10]. Other more frequent sites are chest wall/axilla, shoulder region, inguinal region, buttocks, and neck [11]. In all these cases, preoperative biopsy and histopathological and/or immunohistochemical confirmation of the diagnosis are possible and mandatory. In the previous literature, only few cases of intradural LGFMSs have been described, affecting either frontal lobe, temporal lobe, or CPA [11–14]. We hereby described, to the best of our knowledge, first two cases of extradural LGFMS at the lateral skull base.

There are three main challenges in the management of skull base tumours. First, the preoperative biopsy and histological diagnosis are usually impossible for skull base tumours. Therefore, the diagnosis is established based on clinical findings and radiographic imaging. Neither clinical signs nor radiological findings of the skull base lesions are specific. The lesions can present with various neuropathies of affected cranial nerves (CN) V–XII, tinnitus, hearing loss (HL), and disequilibrium. The location of the tumour, its consistency, infiltrative nature, and its extensions seen on radiologic images are also not diagnosis-specific. The main preoperative diagnosis in our first case was tympanojugular paraganglioma class C2 based on CT and MRI findings. However, typical “salt and pepper” appearance on precontrast T1-weighted sequences, a characteristic for paragangliomas, was not encountered [15]. Therefore, other lateral skull base tumours were also considered, including endolymphatic sac tumours, lower cranial nerve schwannomas, jugular foramen meningiomas, chordomas, and chondrosarcomas [16]. Endolymphatic sac tumour was an alternative diagnosis in our first case. Our assumption was based on the location of the tumour, extending more superiorly than tympanojugular paragangliomas typically do. However, endolymphatic sac tumours are usually centred at the level of the posterior semicircular canal and generally spare jugular foramen [17]. In our first case, the tumour centred at the jugular foramen and from there extended superiorly to erode the posterior semicircular canal. The absence of intratumoral cystic changes, a characteristic for lower cranial nerve schwannomas, and a dural tail, a characteristic for the jugular foramen and all other meningiomas, excluded those tumours [15, 16]. Jugular foramen meningiomas tend to spread medially to involve the jugular tubercle, with further extension anteriorly towards the clivus, in contrast to our case, where the tumour was spreading towards the middle ear and mastoid structures [15]. Chordoma and chondrosarcoma can rarely arise from the jugular foramen and are usually centred at the clivus, which was not a case in our patient [16].

The second challenge in management of skull base tumours is that en bloc resection is impossible in most of the cases. The latter could lead to potentially life-threatening complications due to risk for damaging vital neurovascular structures [18]. Fortunately, the majority of lateral skull base tumours are benign, and piecemeal resection is acceptable. Given that, the decision to keep residual tumour tissue, in order to preserve function of the cranial nerves, is frequently made. Malignancy of the resected tumour is usually identified postoperatively based on the histopathological investigations. Both of our cases are illustrative of the above-mentioned rationale. In our first case, we suggested tympanojugular paraganglioma class based on clinical findings and preoperative imaging and performed an infratemporal fossa approach (IFTA) type A with anterior rerouting of the infiltrated facial nerve for surgical treatment of the disease, thereby preserving its normal function. In our second case, the patient was referred to our department due to recurrence of previously diagnosed LGFMS. We removed large tumour masses separately using the transcochlear approach with selective neck dissection, preserving the part of the tumour infiltrating jugular foramen in order to maintain LCN function. In our opinion, piecemeal resection and leaving behind residual tumour was still acceptable strategy because LGFMSs are slow- growing tumours, the patients have usually long clinical course, and they rarely give regional and distant metastases [3]. Recurrence ranges up to 15 years with a median of 3.5 years, whereas the interval to metastasis varies up to 45 years with a median of 5 years [3]. Surprisingly, LGFMS may have better prognosis than some benign skull base tumours. Jugular foramen meningiomas, for instance, have average postoperative recurrence rate as high as 14% at 2 years [15].

The third challenge is that the histological sample of LGFMS does not usually look malignant, so a special attention should be drawn to recognize a certain histopathological pattern of the tumour. LGFMS is usually relatively hypocellular, composed of uniform, bland short spindle cells, although occasional cases can show variations in cytomorphology and cellularity [8]. Other recognizable morphologic features include prominent hemangiopericytoma- (HPC-) like vasculature, rich networks of curvilinear capillaries, and deposition of keloidal collagen fibers [8]. In both of our cases, the histopathological findings were suggestive of LGFMS (Figure 2).

As the tumour is lacking of atypical features, it has to be distinguished from benign lesions, such as benign nerve sheath neoplasms, perineurioma, myxoma, and fasciitis [19]. Differential diagnosis includes other sarcomas such as myxofibrosarcoma or myxoid dermatofibrosarcoma protuberans [8, 19]. LGFMS is known for its absence of specific immunohistochemical (IHC) markers; although most cases show at least focal expression of EMA, the tumour is usually negative for CD34 and for muscle markers, cytokeratins, and S100 protein [8]. Definite diagnosis can be established on finding evidence of the characteristic translocations for LGFMS by polymerase chain reaction or FISH [8, 19]. Immunohistochemistry for expression of EMA and claudin-1 is usually suggestive on perineurioma [19]. However, both EMA and claudin-1 are rarely positive in LGFMS [19]. MUC4 is a highly sensitive and quite specific IHC marker for LGFMS and can be helpful to distinguish this tumour type from histological mimics, e.g., perineurioma [8].

In our first case, the immunophenotype tests were negative for S100, EMA, SMA, CD34, and Bcl-2 and positive for MUC4 in the cytoplasm. In our second case, the immunophenotype of the sample tissue was negative for S100 and EMA, with focal positivity for MUC4. As both of our patients were young adults and had characteristic histopathologic findings and immunoprofile, the decision was made to diagnose LGFMS.

In both of our cases, the patients received an adjuvant postoperative radiotherapy course. The decision was made in order to control either contaminated resection margins or residual tumour. However, it has been demonstrated that radiotherapy and chemotherapy do not affect the course of the disease with regard to subsequent recurrences, metastases, dedifferentiation, and life expectancy. The reason for low chemo- or radiosensitivity of the LGFMS is connected to its low grade of malignancy and low mitotic rate [20]. However, adjuvant radiotherapy could still be recommended to compensate the difficulty in removing LGFMS within healthy tissue margins [13].

## 5. Conclusions

The lateral skull base is an extremely rare localization of LGFMS. To our best knowledge, we report the first two cases of such an entity. In light of rarity of such LGFMS localizations, each case requires individual treatment approach using different lateral skull base surgery methods. It is potentially life-threatening to perform en bloc resection in lateral skull base surgery approaches, and therefore, the residual tumour is oftentimes preserved in attempt to save important neurovascular structures and their function. Adjuvant radiotherapy may compensate for the difficulty in removing a safety margin of normal tissue in case of LGFMS of intracranial origin. The long-term follow-up is essential in case of LGFMS, as the disease often displays high rate of metastasis.

## Supplementary Materials

Supplementary Materials Supplementary video: the surgical excision of the tumour in our first case performed using infratemporal fossa approach type A (ITFA-A) with preoperative embolization.
